# Strategies to Overcome Antileishmanial Drugs Unresponsiveness

**DOI:** 10.1155/2014/646932

**Published:** 2014-04-30

**Authors:** Shyam Sundar, Anup Singh, Om Prakash Singh

**Affiliations:** Department of Medicine, Institute of Medical Sciences, Banaras Hindu University, Varanasi 221005, India

## Abstract

In the absence of effective vector control measures and vaccines against leishmaniasis, effective chemotherapy remains the mainstay of treatment. As the armoury of antileishmanial drugs is limited, strategies should be made to target the emergence of drug resistance. The loss of efficacy of antimonials such as sodium stibogluconate in the Indian subcontinent which has been the mainstay of treatment for more than six decades has raised concern to save the other drugs. In the current review, we highlight various steps which could be implemented to halt the increasing unresponsiveness of drugs such as monitoring of therapy in the form of rational dosing and duration of treatment, understanding the mechanism of action of the drugs and drug resistance, identification of markers of resistance, distribution of drugs free of cost, evolution of effective combination therapy and immunotherapy, and proper management of HIV/VL coinfection and post-kala-azar dermal leishmaniasis (PKDL). Strong support from governmental agencies and local communities in the form of education and orientation programmes for feasibility of implementing these strategies and affordability within the context of their health systems is needed in controlling and preventing leishmaniasis.

## 1. Introduction

In view of limited armamentarium of antileishmanial drugs, it is imperative that effective monitoring of antileishmanial drug use be done to prevent the emergence of resistance. Visceral leishmaniasis elimination programme was launched by three countries, India, Nepal, and Bangladesh, with an aim to reduce the incidence of visceral leishmaniasis (VL) to <1 per 10,000 population in the Indian subcontinent by 2015 [1]. However, emergence of antileishmanial drug unresponsiveness, relapse cases, and drug resistance makes its management and hence elimination challenging. Sodium stibogluconate (SSG), a pentavalent antimonial compound, remains the treatment of choice in Africa, South America, Bangladesh, Nepal, and India (except North Bihar) at the dose of 20 mg/kg/day parenterally for 28–30 days. In North Bihar the cure rate has dropped to 64% and even lower in subsequent studies [2]. The reasons for the emergence of resistance were the widespread misuse of the drug. SSG was prescribed in inappropriate doses and duration by both qualified medical practitioners and unqualified quacks which led to its widespread misuse. Almost 73% of patients consulted unqualified practitioners first; most of them did not use the drug appropriately [2, 3]. In order to minimize the toxicity, especially nephrotoxicity, it was a common practice to start with a small dose and gradually escalate to the full dose over a week, advocating drug free periods and splitting of the daily dose into two injections. These malpractices eventually resulted in its subtherapeutic blood levels and increased tolerability of parasites to SSG. HIV/VL coinfected patients are another subset who respond poorly to SSG, as an intact immune system is required for its response. Initial parasitological cure could be as low as 37% with SSG as most of them tend to relapse [3]. Thus, they are a potential source for emergence of drug resistance.

Amphotericin B, a polyene antibiotic, is now being used as a first line therapy in areas with SSG resistance. It has excellent cure rates (~100%) at doses of 0.75–1.00 mg/kg for 15 infusions daily or on alternate days. It has been used extensively in Bihar with uniformly good results [4, 5]. Lipid formulations of amphotericin B are as effective as conventional amphotericin B and have negligible adverse reactions. The dose requirement of liposomal amphotericin B varies from region to region, while in the Indian subcontinent a small dose induces high cure rates, whereas a higher dose is needed for Eastern Africa, the Mediterranean region, and Brazil [6–8]. Clinical resistance to amphotericin B is rare. Nevertheless, with the increasing use of amphotericin B, especially in lipid formulations which have longer half-life, there could be possibilities of resistance associated with the drug.

Miltefosine, an alkyl phospholipid, is the first oral agent approved for the treatment of leishmaniasis. At the recommended doses (100 mg daily for patients weighing ≥25 kg and 50 mg daily for those weighing ≤25 kg for 4 weeks) cure rates were 94% for VL [9]. However, it is associated with frequent gastrointestinal adverse events, teratogenic potential and long half life (~1 week) which renders it vulnerable to resistance. Patients tend to discontinue drug prematurely due to side effects if not counselled beforehand. Quick recovery within a few days of the start of therapy, which may lead to premature discontinuation, is also a concern. In these patients with incomplete treatment, its long half-life will lead to persistence of subtherapeutic levels of the drug along with the parasites, leading to tolerance and drug resistance, and in anthroponotic foci like the Indian subcontinent, this will lead to exponential rise in these refractory parasites [10]. In a recent study done in North Bihar it was observed that the relapse rate doubled with miltefosine within 10 years since its introduction [11]. The pharmacokinetics and pharmacodynamic relationship between VL treatment and miltefosine exposure has been recently explored; it was reported that children are less exposed to miltefosine than adults under conventional chemotherapy and are thus at high risk of treatment failure [12, 13].

Sitamaquine (8-aminoquinoline) is the second oral drug discovered by the Walter Reed Army Institute of Research (WRAIR, USA) and in development with GlaxoSmithKline (UK) for the oral treatment of VL. It is an active drug with short elimination half-life (about 26 hrs) and appears to be a promising agent for treatment of VL both in India [14] and in Africa [15]. Its bioavailability is better than those of miltefosine but a major disadvantage is safety of the drug, mainly at the level of methemoglobinemia and nephrotoxicity.

Paromomycin has been recently introduced for VL treatment in mono- as well as combination therapy. After a phase III trial of paromomycin, an aminoglycoside-aminocyclitol antibiotic, in the Indian subcontinent, which showed it to be noninferior to amphotericin B, it was approved by the Indian government in August 2006 for the treatment of patients with visceral leishmaniasis and is recommended by WHO [16]. Clinical resistance with this drug in VL is not known as it has not been used extensively.

Apart from these available antileishmanial drugs, there are three new drugs currently under the pipeline of DNDi for treatment of leishmaniasis [17].

Importantly and relevant to this discussion, drug therapy works most effectively with help from the host immune system, and response to treatment varies between geographical regions, requiring higher dose of drug in East Africa than in South Asia. Further, the standard treatment for VL in East Africa still consists of antimonials; however, unresponsiveness to this drug is common in the Indian subcontinent [18–20].

As we see the sparse inventory of antileishmanial drug and the emergence of resistance, it is high time we implement strategies which could overcome drug unresponsiveness. The following steps could be taken.

### 1.1. Proper Monitoring of the Treatment to Ensure Compliance and Treatment Protocol

Routine monitoring of antileishmanial drug responsiveness is essential for the success of the* Leishmania* elimination programme as any resistance could result in an outbreak of diseases given the low or absent immunity to* Leishmania* within the population. Theoretically, a failing drug that does not clear parasitemia further increases the infective reservoir and could lead to increased VL transmission and VL epidemics in areas with previously low transmission. However, it is difficult to attribute the occurrence of an epidemic due to decreasing efficacy of a drug but there are a few reports from other diseases like malaria, where occurrence of a malaria epidemic was identified due to drug resistance as the major reason [21]. According to a study conducted to detect the factors leading to antimony resistance in Indian VL, it was observed that WHO guidelines were followed in only 26% of patients, 42% of the drugs were not taken regularly, and 36% of the patients stopped the drug on their own will [22]. Similar concerns were raised for miltefosine when preliminary data from a phase IV trial in India involving domiciliary treatment with miltefosine and weekly supervision showed doubling of the relapse rate [23]. These findings suggest that monitoring therapy is essential to prevent development of resistance. The directly observed treatment strategy (DOTS) for tuberculosis has been a big success and either a parallel or integrated strategy with DOTS system could be enforced for leishmaniasis. WHO recommends DOTS for miltefosine [24]. This will ensure not only better compliance but also appropriate completion of the treatment course and will ultimately prevent resistance.

### 1.2. Availability of Drugs Free of Cost with Proper Practice and Efficient Health System

The high cost of the antileishmanial drugs coupled with over-the-counter availability often leads to underdosing and incomplete treatment, fostering the selection of secondary resistant parasites, and therefore has a higher likelihood of treatment failure. It is therefore important that antileishmanials should be made available free of cost and should be distributed through public and/or private health care providers like antitubercular and antiretroviral drugs considering the fact that the majority of the patients cannot afford to purchase and complete a full course of treatment. Although important, mere provision of “free supply” of drugs will not decrease resistance, but a proper practice and health system in dispensing drugs should be enforced because as in the case of other infectious diseases like tuberculosis, malaria, and so forth, despite free supply of drugs, data on decreased resistance is inconsistent. Furthermore, the availability of the drug should be barred from open market. This has been the major factor for antimony resistance and could lead to resistance to other drugs as well especially the novel oral agent miltefosine which is the basis of VL elimination programme. Moreover, private as well as government medical practitioners should be warned about not giving treatment before confirmation through a laboratory or biological test. Some still give it after a physical examination of body temperature and an enlarged spleen. Therefore, treating with the recommended drugs, taking medicine only after diagnosis, and following prescriptions will go a long way to save antileishmanial drugs, ensuring effective treatment.

### 1.3. Encouraging Multidrug Therapy

Multidrug combination therapy has been used successfully in tuberculosis, leprosy, and malaria. The rationale behind combination therapy is to increase the activity through use of compounds with synergistic or additive activity, prevent emergence of drug resistance, lower dose requirement thereby reducing chances of toxic side effects and cost. The growing resistance of the parasite to antileishmanial drugs suggests that the currently used monotherapy needs to be reviewed. Various trials have shown combination therapy to be as effective as conventional regimen and is now recommended by WHO for Indian VL [25, 26]. The inventory of the antileishmanial drug is limited; thus it is of utmost importance to save and prolong the effective life of the existing drugs. Also, it is important to mention that even for combination therapies the treatment should be directly observed to ensure compliance and decrease drug resistance to each of the drugs in combination regimen.

### 1.4. Understanding Mechanism of Drug Resistance and Identification of Markers of Resistance

Clinical resistance and therapeutic failures in VL patients have been increasingly recognized in recent years, leading to the need for innovative and alternative therapies against leishmaniasis. The way drug resistance is emerging and spreading is not at all understood. Nothing is known about the dynamics of drug resistant* Leishmania* populations in the presence or absence of drug pressure. Although dormancy during antileishmanial treatment has not been demonstrated in laboratory studies, researchers suggest that small numbers of inactive parasites could survive these exposures and later reactivate with the same characteristics of drug sensitivity as the original population. Therefore, surveillance for phenotypic and genotypic markers of resistance should be done in clinical isolates at regular intervals. Primary resistance of the parasite should be studied rather than relapses or unresponsiveness. It will help in identification of not only key intracellular targets but also the parasites defence mechanisms. This can be used to design rational analogues of existing drugs that would overcome the existing defence mechanism of most parasites. At present there are no molecular markers of resistance available for the currently used antileishmanial drugs and the only reliable method for monitoring resistance of isolates is the in vitro amastigote-macrophage model which is technically demanding. To identify markers for in vitro SSG-resistance in clinical* L. donovani* isolates by targeting the thiol metabolism of the parasite, important for activation of the drug and defense against its action mechanism, and import/export mechanisms for the drug, several studies have been done [27]. However, these candidate markers have yet to be validated on a larger sample or were not uniformly present in all SSG-resistance strains. Genetic markers of resistance have also been explored but are not generalised irrespective of the endemicity [28]. Studies to find the markers of resistance to miltefosine which is the backbone of VL elimination programme are underway and discussed elsewhere [29–31]. Resistance to amphotericin B and paromomycin has not been studied apart from a few reports. Understanding the molecular and cellular mechanism of drug resistance will lead to the development and deployment of novel therapeutic strategies with the potential to sensitize chemotherapy and to increase the efficacy of current treatments in a wide variety of human leishmaniasis. Drug resistance markers, once identified, can be applied as an efficient tool for resistance surveillance.

### 1.5. Understanding the Drug Response Variability in Patients at Genetics Level

Treatment outcome is a complex phenomenon that essentially depends on the interaction between the drug, the host, and the parasite. For example, in Bihar, it seems that parasite resistance is consistently correlated with SSG treatment failure [32, 33]. In contrast, in Nepal VL patients infected with SSG resistant parasites only have about 25% risk for SSG treatment failure [34]. The differences between the 2 areas could be due to biological differences between the parasite populations (like different stages in the acquisition of resistance) or due to ethnic differences. The Clinical Pharmacogenetics Implementation Consortium (CPIC) has been providing the information on how genetic test results can be used to optimize drug therapy since 2009 [35]. Pharmacogenetics is the study of how interindividual variations in the DNA sequence of specific genes affect drug response. Differences in drug response can be attributed to variability in DNA sequences of specific genes, products of which are crucial for drug metabolism. The cytochrome P450 (CYP) enzymes have been identified as major players in drug metabolism and human genome project identified 57 human CYPs which play an important role in the metabolism of therapeutic drugs, other xenobiotics, and some endogenous compounds [36]. CYP2D6, 2C19, and 2C9 polymorphisms account for the most frequent variations in phase I metabolism of drugs, since almost 80% of drugs in use today are metabolized by these enzymes.

Once genetic testing becomes part of routine analysis, such information will enable prediction of complications in drug therapy and development of a personalized treatment regimen, where drug dosages are based on an individual's specific CYP profile [37]. Ultimately, this approach offers a better treatment option by preventing treatment failures and avoids unnecessary side effects. Another interesting approach could be the consideration of CYP polymorphisms in clinical trials. Potentially, it would decrease the failures if information of potential polymorphisms in different ethnic groups is included. Several studies emphasize the importance of CYP polymorphisms and alternatively metabolized drugs in clinical practice. Prediction of CYP activity could be helpful to assess drug response.

### 1.6. Promoting Research on Vaccine Development

Despite advances in chemotherapeutic options, increasing unresponsiveness makes the management of disease complicated. It is unlikely that chemotherapy alone will enable disease elimination. Vaccine is the only option to break the life cycle of parasite by boosting host immune response. The genome sequence of* Leishmania* has offered a new way to understand the pathogenesis and host immunological status caused by different* Leishmania* species, suggesting that it may be possible to generate broadly effective vaccines against different clinical diseases. Significant progress has been made in the past years to identify recombinant antigens that can protect against* Leishmania* infection in experimental models. Some of these antigens include amastigote specific proteins A2 [38], kinetoplastid membrane protein-11 [39], cysteine proteinase B [40],* Leishmania* homolog of the receptor for activated C kinase (LACK) [41], tryparedoxin peroxidase (TRYP) [42], promastigote surface and surface expressed glycoprotein gp 63 [43], and autoclaved whole* Leishmania* parasite antigen [44]. Although most of these recombinant antigens have shown immunogenicity and protective efficacy in animal models, only a few have progressed to clinical trials in preclinical human studies [45, 46]. A multisubunit recombinant* Leishmania* vaccine, Leish-111F, containing an* L. major* homolog of eukaryotic thiol-specific antioxidant,* L. major* stress inducible protein-1, and* L. braziliensis* elongation and initiation factor, in formulation with MPL-SE, has been shown to provide protection in Indian people with or without prior VL exposure [47]. The efficacy of this vaccine is modest, but it could make a useful contribution to reducing VL in high burden settings and in elimination settings.

### 1.7. Proper Management of HIV/VL Coinfection

HIV changes the nature of the human VL infection, the response to treatment, and the epidemiology. The HIV/VL coinfected patients have a high parasite burden and a weak immune response, respond poorly to treatment, and have a high relapse rate. Therefore, they are the ideal candidates to harbour and spread drug resistant parasites. With the growing burden of HIV in India, HIV/VL coinfection could become a major problem. Experience from Southern Europe shows that initial response to SSG and conventional amphotericin B is low (~40–65%) in severely immunocompromised persons and severe adverse events are frequent. Therefore, the only effective drug in HIV/VL coinfection is Ambisome [48]. Incidence of VL coinfection dramatically decreases by initiation of HAART. Therefore, HAART in combination with antileishmanials should be advocated strictly in these patients.

### 1.8. Training and Medical Education Programme

For maximal benefit from the current effective* Leishmania* prevention and control programmes, it is very important that local communities should be educated to enable them to make informed decisions. Prevention and control of* Leishmania* whether by use of bed-nets or indoor residual spray (IRS), environmental management to prevent sand fly breeding sites, or promoting early diagnosis and correct treatment require constant involvement of community. One of the major reasons for antimony resistance was lack of awareness among the affected population and health care providers about the need for effective treatment and control of kala-azar. There is a considerable need and scope for orientation programs to educate those at risk, doctors, and the government agencies responsible for controlling and preventing kala-azar in India.

### 1.9. Surveillance of Post-Kala-Azar Dermal Leishmaniasis (PKDL) Cases Detection and Its Effective Management

Treatment of PKDL is an important and unresolved issue which needs to be addressed for achievement of the visceral leishmaniasis elimination programme which aims to eliminate PKDL by 2018. PKDL cases serve as a reservoir of* Leishmania* infection in the population and should be diagnosed and treated effectively. WHO has recommended various regimens for PKDL based on geographical distribution but each has a long course and is associated with toxicities [24]. In India cure rates are 64–92% with sodium stibogluconate (SSG) in doses of 20 mg/kg per day for 120 days [49]. Similarly three 20-day infusions of amphotericin B for an interval of 20 days pose a real threat of nephrotoxicity [50]. These long parenteral regimens invariably lead to either nonacceptance or poor compliance. Ambisome (liposomal amphotericin B) in the dose of 2.5 mg/kg for 20 injections is another alternative with minimal side effects but due to its high cost, it is not a feasible option [51]. Oral miltefosine has been successfully used in doses of 100 mg/day for 12-week and 8-week regimen and found to be an effective alternative for treatment of PKDL with a cure rate of 93 and 81%, respectively [52]. However, paromomycin has failed to show its efficacy when used in a dose of 11 mg/kg for 45 days in Bihar, India (unpublished data). As few cases of PKDL are enough to maintain the reservoir of infection and with limited ineffective treatment option, apart from proper case finding tools, efficacy of combination therapy should be explored to decrease the treatment duration, increase compliance, and to curtail the infection in the population. A passive surveillance for PKDL in the public sector is further hampered by an unregulated private sector which fails to notify PKDL disease even where required by regulation.

### 1.10. Interrupting Parasite Life Cycle by Vector Control Programme

Interruption of the parasite life cycle in which new (resistant and multidrug resistant) genotypes emerge by vector control may offer a cheaper, more practical solution to drug unresponsiveness. In the context of the vector control, the female* Phlebotomus argentipes* should be considered as the moving target and thus control may be broadly divided into three main categories: (i) reducing vector density, (ii) interrupting life cycle, and (iii) creating a barrier between the human host and the sand fly vector. However, the reduction in parasite transmission and subsequently in VL incidence by effective vector control is still an open question. Evidence from Sudan was encouraging: it showed a reduction in VL incidence through mass distribution of long lasting insecticide nets (LNs) [53, 54] and also provided some degree of protection against* L. donovani* infection in India [55]. To date reports of resistance to insecticide (DDT) to three species (*Phlebotomus papatasi*,* P. argentipes, and Sergentomyia shorti*) are found only in India [56], although there are reports of increased tolerance to this compound in several countries. Therefore, vector control measures such as insecticide treated bed-nets and other personal protective measures need to be encouraged rather than spraying DDT alone as indoor residual spray. There are many lessons to be learned from malaria control programme including the realization that the aim of sand fly control should not only be to reduce the relative abundance of a vector species, but also to prevent the sand fly bite by reducing the contact of its blood feeding females with humans and to reduce their life spans so that fewer survive long enough to transmit* Leishmania* parasites.

### 1.11. Improving the Efficacy of Treatment by Immunotherapy

Over the last two decades, immune based therapies, either alone or combined with chemotherapy, have been developed for better treatment and for preventing drug resistance in leishmaniasis [57]. Studies indicate that certain strategies aimed at modulating the host immune response by use of biological substances could result in improvement of therapeutic cure of leishmaniasis under both laboratory and field conditions. The use of biological molecules or compounds to modulate or modify immune responses in order to achieve the prophylactic or therapeutic goal has been tested in both preclinical and clinical studies in treatment of leishmaniasis [58]. Immunotherapy with recombinant human IFN*γ* and pentavalent antimonials is reported as a stronger parasitologic and clinical cure compared with the drug alone in VL patients from Brazil, Kenya, and India [59–61]. Whatever the approach, it is increasingly becoming apparent that the most promising chemotherapies cannot work alone. Chemotherapies and immunotherapies should be combined effectively to attack the parasite from multiple sides to quickly and thoroughly eliminate the disease. Thus, the use of a combination therapy and targeted therapies could aid in addressing some of the current challenges associated with the management and treatment of VL, namely, minimizing resistance to currently available drugs, improving the therapeutic index, decreasing the dose or length of treatment, reducing the cost of therapy, and improving adherence to treatment.

## 2. Conclusion

Treatment option of VL is limited, and emergence of drug resistance is further complicating the control of leishmaniasis. To deal with drug resistance, the most severely affected countries need to select the best affordable treatment option as quickly and cautiously as possible so as to avert deaths and also protect the drugs from the development of resistance.

Changes in the drug policy are a much needed step as of today; however, the effective implementation of the new drug regimen (e.g., oral miltefosine and Ambisome) at ground level should be encouraged. Improving health infrastructure at a peripheral level is of the top most importance for improvement in disease status in the endemic areas. Regular monitoring of drug resistance and associated molecular markers is needed to be studied at a larger extent for proper inputs to the drug policy makers. Understanding the mechanism of action of the drugs and mechanisms involved in drug resistance needs to be explored for designing a better and effective drug regimen against VL. Combination chemotherapy and single dose liposomal amphotericin B have been recommended by WHO and should be encouraged. Apart from free availability of drugs and directly observed treatment for ensuring compliance, education about the disease and importance of complete treatment is essential to constrain the disease. Likewise, vector research should speed up the development of new insecticides to replace the failing synthetic DDT and to create sand flies refractory to* Leishmania* transmission. More resources are required, and collaboration with both public/private sectors and across borders is essential.
